# Side Effects of COVID-19 Vaccines Among Diabetic Subjects and Healthy Individuals

**DOI:** 10.7759/cureus.36005

**Published:** 2023-03-10

**Authors:** Fareena Khan, Muhammad Taimur Khan, Sana Zaman, Sadaf Mujtaba, Aeliya Batool, Zohra Ghanghro, Adnan Anwar, Atif A Hashmi

**Affiliations:** 1 Anaesthesia, Baqai Medical University, Karachi, PAK; 2 Family Medicine, Standish Medical Practice, Standish, GBR; 3 Family Medicine, Alastair Ross Medical Practice, Bolton, GBR; 4 Pharmacology and Therapeutics, Hamza Medical Center and Pharmacy, Karachi, PAK; 5 Pharmacology, Karachi Medical and Dental College, Karachi, PAK; 6 Paediatrics, South City Hospital, Karachi, PAK; 7 Physiology, Hamdard College of Medicine and Dentistry, Karachi, PAK; 8 Pathology, Liaquat National Hospital and Medical College, Karachi, PAK

**Keywords:** fever, pain, side effects, diabetes, vaccines, covid-19

## Abstract

Objectives

Vaccinations protect against disease through various ways, but the process of developing immunity might result in side effects. This study determined the immediate side effects of coronavirus disease 2019 (COVID-19) vaccination among patients with diabetes and non-diabetic participants.

Methods

This multi-center, cross-sectional study was conducted in multiple hospitals in Karachi, Pakistan. The duration of the study was six months, from March 1, 2022, to August 31, 2022. A total of 1150 participants who received one of the COVID-19 vaccines, for instance, Sinopharm, AstraZeneca, Sinovac, and Pfizer (double or booster doses) were included in the study and allocated into two groups: diabetics (n=540) and non-diabetics (n=610). The chi-square test was used to compare the frequency of side effects between diabetic and non-diabetic participants. The association between means of demographic variables such as age and weight was compared using an independent t-test.

Results

The study findings showed that the mean age of the group with diabetes was 46.73±14.98 years and that of the non-diabetic group was 44.01±14.80 years with a significant difference between them (p=0.002). The majority of participants, 187 (34.6%) patients with diabetes received Pfizer, while 234 (38.4%) non-diabetic participants received Sinovac. Following the first dose, side effects were higher in patients with diabetes. Burning at the injection site, fever, and pain were the most commonly reported side effects in patients with diabetes following the first dose. Additionally, following the second dose, burning at the injection site, muscular pain, fever, and pain were the most commonly observed side effects, with a significant association among diabetic and non-diabetic participants (p<0.001).

Conclusion

Diabetes is a prevalent comorbidity in individuals infected with COVID-19, and patients with diabetes report more side effects from immunization than non-diabetic participants. The most commonly reported side effects of the vaccine in diabetic participants were observed to be burning at the injection site, fever, muscle and joint pain, and swelling at the injection site. Additionally, participants with and without diabetes reported feeling satisfied with their vaccines.

## Introduction

Over 100 million people worldwide have been infected with the novel coronavirus disease 2019 (COVID-19), which has also resulted in over two million fatalities. A bad prognosis is predicted by the illness, especially for those who have diabetes mellitus (DM). In patients with COVID-19, DM is linked to severe illness, intensive care unit (ICU) hospitalizations, and higher deaths [1-3].

Recent research has demonstrated that patients with type 2 diabetes (T2D) and type 1 diabetes (T1D) are more susceptible than those without DM to developing serious sickness from COVID-19. Patients with T1D and T2D had comparable adjusted odds ratios (ORs) for hospitalization (3.90 for T1D vs. 3.36 for T2D), disease severity (3.35 vs. 3.42), and in-hospital fatality (3.51 vs. 2.02) [4,5]. In addition, improved outcomes in patients with DM hospitalized for COVID-19 have not been consistently linked to adequate glycemic control before hospital admission, as shown by glycated hemoglobin (HbA1c) [6]. Therefore, primary prevention continues to be the cornerstone of reducing COVID-19 risks in individuals with DM.

DM patients are almost three times more likely to die from COVID-19 and are more likely to experience severe symptoms following viral infection. Because they frequently have more severe consequences than persons without DM, patients with DM are given priority for early vaccines [7]. To prevent COVID-19 infection, numerous vaccines have been authorized [8,9]. According to reports, the vaccines offered by Johnson & Johnson, Pfizer, Moderna, and BioNTech are efficient and secure for people with diabetes. The Oxford/AstraZeneca vaccine has been associated with extremely uncommon side effects, such as blood clot development. However, the advantages of this vaccine surpass the dangers of blood clot formation in diabetics [7,10]. People who received COVID-19 immunization typically have only minimal side effects. Additionally, after receiving the COVID-19 vaccine, patients with DM may develop moderate side effects such as a low-grade fever, discomfort, redness, or swelling around the injection site. People with DM only seldom experienced serious adverse responses after receiving the COVID-19 vaccination [7].

An important first step in protecting individuals with DM against the dangers associated with COVID-19 is timely and proper vaccination. Global efforts to create severe acute respiratory syndrome coronavirus 2 (SARS‑CoV‑2) vaccines have been ongoing since the start of the COVID-19 pandemic. Numerous vaccinations have been developed so far, including mRNA vaccines such as mRNA-1273 and BNT162b2, vector-based vaccines such as AZD1222 (ChAdOx1), inactivated viral vaccines such as CoronaVac and COVAXIN (BBV152), and Sputnik V vaccines including GamCOVID-Vac and JNJ-78436735 [11]. Although the effectiveness of COVID-19 vaccinations has been evaluated in the general population, including in patients with DM, subgroup analysis has primarily been performed among high -risk patients in general rather than patients with DM in particular [12]. There is still some controversy around the use of COVID-19 vaccinations in individuals with diabetes.

In people with DM, adverse reactions to immunization are typically mild. A total of 1568 DM patients (duration more than 5.3 years) at an Indian DM clinic received influenza and pneumonia vaccines. The only adverse effects that were noticed were fever, a localized rash, enlarged glands, and joint or muscle pain. There were no incidents of serious allergic responses. Only 17 of 2057 patients with DM who received pneumococcal vaccines in a study by Kesavadev et al. reported mild pain or redness around the injection site [13].

The most recent recommendations or guidelines for SARS-CoV-2 vaccination in patients with DM differ from country to country. The percentage of DM patients who were reluctant to get vaccinated ranged from 14.2% in Italy, 24.7% in Malaysia, to 29.0% in Saudi Arabia due to a lack of statistical evidence supporting the benefits of immunization [14-16]. Previous research on vaccine hesitancy in DM patients has mostly looked at high-income nations; little is known about the attitudes currently prevalent in low- and middle-income nations. According to a survey done in Uganda, 29.90% of people with chronic conditions, such as DM, were reluctant to obtain the SARS-CoV-2 vaccine [17]. Uncertainties about the vaccine's components and worries about its negative effects are the main causes of vaccine reluctance among diabetics [14-16].

There is currently limited data on the prevalence of chronic diseases like DM in developing countries like Pakistan. Furthermore, it is critical that physicians are aware of any possible side effects or complications in patients with DM before getting COVID-19 vaccines. This study compared patients with and without DM who received the first and second doses of the COVID-19 vaccination to identify the prevalence and development of side effects.

## Materials and methods

This multi-center, cross-sectional study was conducted in multiple hospitals in Karachi, Pakistan. The non-probability sampling technique was employed in this study. The duration of the study was six months, from March 1, 2022, to August 31, 2022. A total of 1150 participants who received one of the COVID-19 vaccines, for instance, Sinopharm, Astrazeneca, Sinovac, and Pfizer (double or booster doses), were included in the study and were allocated into two groups: diabetics (n=540) and normal or non-diabetics (n=610). Those who were not vaccinated were excluded from the study.

Information about the participants was gathered via a self-designed questionnaire. Demographic details of participants such as age, gender, co-occurring diseases, vaccination type and doses, prior exposure to COVID-19 infection, and the incidence of any general and local side effects following first- and second-dose vaccination were all recorded. The degree of satisfaction among the participants was also recorded. If a participant's HbA1c level was more than 6.5%, they were deemed diabetic. The remaining participants who had normal blood sugar levels and had never taken home anti-diabetic medications in the past made up the non-diabetic group.

Data analysis was performed using IBM SPSS Statistics for Windows, Version 26.0 (Released 2019; IBM Corp., Armonk, New York, United States). Different demographic parameters (gender, vaccine type, number of doses, and local and systemic adverse effects) were computed as frequencies and percentages. The chi-square test was used to compare the frequency of side effects between diabetics and non-diabetics. The association between means of demographic variables such as age and weight was compared using an independent t-test. A p-value of < 0.05 was considered statistically significant.

## Results

A total of 1150 participants who were vaccinated against COVID-19 were included in the study, of which 540 (46.9%) had DM and 610 (53.04%) were non-DM. In the group with DM patients, 337 (62.4%) were males and 203 (37.6%) were females, whereas in the non-DM group, 297 (48.7%) were males and 313 (51.3%) were females. The mean age of the DM group was 46.73±14.98 years and the mean age of the non-DM group was 44.01±14.80 years with a significant difference between them (p=0.002). The mean weight of the DM group was 72.21±14.81 kg and the mean weight of the non-DM group was 69.05±17.36 kg with a significant difference between them (p=0.001). Moreover, 319 (59.1%) had hypertension in the DM group and 279 (45.7%) had hypertension in the non-DM group, with a significant association between them (p<0.001).

A total of 263 (48.7%) in the DM group had been infected with COVID-19 as opposed to 78 (12.8%) in the non-DM group, with a significant association (p<0.001). Additionally, 66 (12.2%) in the DM group and 26 (4.3%) in the non-DM group had previous exposure to COVID-19 infection, with a significant association between them (p<0.001). In terms of the type of injected vaccine, Sinopharm was received by 116 (21.5%) patients with DM and 150 (24.6%) non-DM participants. Sinovac was received by 137 (25.4%) patients with DM and 234 (38.4%) non-DM participants. AstraZeneca was received by 100 (18.6%) DM patients and 111 (18.2%) non-DM participants. Pfizer was received by 187 (34.6%) patients with DM and 115 (18.7%) non-DM participants, with a significant association observed between all vaccines and both groups (p<0.001). Approximately, 460 (85.2%) patients with DM and 502 (82.3%) non-DM participants received the first and second dose of the vaccine, whereas 80 (14.8%) DM patients and 108 (17.7%) non-DM participants received the first and second dose along with a booster dose of vaccine, although there was a significant association observed among them (p<0.001), as shown in Table 1. 

**Table 1:** The basic demographic characteristics of DM patients and non-DM participants (n=1150) SD: standard deviation; DM: diabetes mellitus; COVID-19: coronavirus 2019

Variables		DM Group	Non-DM Group	p-value
Age (years), mean±SD		46.73±14.98	44.01±14.80	0.002
Weight (kg), mean±SD		72.21±14.81	69.05±17.36	0.001
Gender, n (%)	Male	337 (62.4%)	297 (48.7%)	<0.001
	Female	203 (37.6%)	313 (51.3%)	
Hypertension, n (%)	Yes	319 (59.1%)	279 (45.7%)	<0.001
	No	221 (40.9%)	331 (54.3%)	
COVID-19 Infection, n (%)	Yes	263 (48.7%)	78 (12.8%)	<0.001
	No	277 (51.3%)	532 (87.2%)	
Previous COVID-19 Exposure	Yes	66 (12.2%)	26 (4.3%)	<0.001
	No	474 (87.8%)	584 (95.7%)	
Type of Vaccine, n (%)	Sinopharm	116 (21.5%)	150 (24.6%)	<0.001
	Sinovac	137 (25.4%)	234 (38.4%)	
	AstraZeneca	100 (18.6%)	111 (18.2%)	
	Pfizer	187 (34.6%)	115 (18.7%)	
Vaccination Status, n (%)	Vaccinated with 1^st^ and 2^nd^ dose	460 (85.2%)	502 (82.3%)	<0.001
	Vaccinated with Booster Dose	80 (14.8%)	108 (17.7%)	

Following the first dose, the risk of side effects was higher in patients with DM. Burning at the injection site (n=455, 84.3%) followed by fever (n=385, 71.3%) were the most commonly observed side effects in DM patients, with a significant difference observed between the DM and non-DM groups (p<0.001). Pain at the injection site was experienced by 349 (64.6%) DM and 284 (46.6%) non-DM participants, with a significant difference observed among them (p<0.001). Furthermore, there was a significant association observed between the DM and non-DM groups in terms of swelling at the injection site, redness at the injection site, lymphadenopathy, headache, nausea, rashes, flu, anxiety, muscle pain, fatigue, joint pain, chills, cough, swelling of the glands, sore throat, shortness of breath, diarrhea, and chest pain (p<0.001), as shown in Table 2.

**Table 2:** The distribution of side effects after first dose of COVID-19 vaccine in the DM and non-DM groups DM: diabetes mellitus; COVID-19: coronavirus disease 2019

Variables	DM Group		Non-DM Group		p-value
	Yes, n (%)	No, n (%)	Yes, n (%)	No, n (%)	
Pain at injection site	349 (64.6%)	191 (35.4%)	284 (46.6%)	326 (53.4%)	<0.001
Swelling at injection site	363 (67.2%)	177 (32.8%)	220 (36.1%)	390 (63.9%)	<0.001
Redness at injection site	160 (29.6%)	380 (70.4%)	118 (19.3%)	492 (80.7%)	<0.001
Lymphadenopathy	296 (54.8%)	244 (45.2%)	117 (19.2%)	493 (80.8%)	<0.001
Fever (temperature (>37.8 ˚C)	385 (71.3%)	155 (28.7%)	359 (58.9%)	251 (41.1%)	<0.001
Headache	263 (48.7%)	277(51.3%)	124 (20.3%)	486 (79.7%)	<0.001
Nausea	150 (27.8%)	390 (72.2%)	58 (9.5%)	552 (90.5%)	<0.001
Rashes	307 (56.9%)	233 (43.1%)	132 (21.6%)	478 (78.4%)	<0.001
Burning at injection site	455 (84.3%)	85 (15.7%)	252 (41.3%)	358 (58.7%)	<0.001
Flu	171 (31.7%)	369(68.3%)	70(11.5%)	540(88.5%)	<0.001
Anxiety	207 (38.3%)	333 (61.7%)	152(24.9%)	458 (75.1%)	<0.001
Muscle pain (myalgia)	374 (69.3%)	166 (30.7%)	136 (22.3%)	474 (77.7%)	<0.001
Fatigue	190 (35.2%)	350 (64.8%)	166 (27.2%)	444 (72.8%)	0.004
Joint pain	364 (67.4%)	176 (32.6%)	174 (28.5%)	436 (71.5%)	<0.001
Chills	316 (58.5%)	224 (41.5%)	176 (28.9%)	434 (71.1%)	<0.001
Cough	269 (49.8%)	271 (50.2%)	108 (17.7%)	502 (82.3%)	<0.001
Swelling of glands	167 (30.9%)	373 (69.1%)	139 (22.8%)	47 1(77.2%)	0.002
Sore throat	276 (51.1%)	264 (48.9%)	145 (23.8%)	465 (76.2%)	<0.001
Shortness of breath	223 (41.3%)	317 (58.7%)	169 (27.7%)	441 (72.3%)	<0.001
Diarrhea	217 (40.2%)	323 (59.8%)	79 (13.0%)	531 (87.0%)	<0.001
Chest pain	167 (30.9%)	373 (69.1%)	119 (19.5%)	491 80.5%)	<0.001

The distribution of side effects after the second dose of the COVID-19 vaccine among the DM and non-DM groups revealed that the most commonly observed side effect was burning at the injection site, which was felt by 388 (71.9%) DM patients and 192 (31.5%) non-DM participants, with a significant difference between groups (p<0.001). Furthermore, muscular pain was reported by 361 (66.9%) DM patients and 178 (29.2%) non-DM participants, with a significant difference observed among them (p<0.001). Pain at the injection site was experienced by 368 (68.1%) in the DM group and 226 (37.0%) in the non-diabetic group, with a significant difference observed among them (p<0.001). Additionally, swelling at the injection site, lymphadenopathy, fever, headache, nausea, rash flu, anxiety, fatigue, joint pain, chills, diarrhea, cough, sore throat, swelling of the glands, shortness of breath, and chest pain were significantly associated with both groups (p<0.001). On the other hand, an insignificant association was observed between redness at the injection site in both groups (p=0.075), as shown in Table 3.

**Table 3:** The distribution of side effects after second dose of COVID-19 vaccine in the DM and non-DM groups DM: diabetes mellitus; COVID-19: coronavirus disease 2019

Variables	DM Group		Non-DM Group		p-value
	Yes, n (%)	No, n (%)	Yes, n (%)	No, n (%)	
Pain at injection site	368 (68.1%)	172 (31.9%)	226 (37.0%)	384 (63.0%)	<0.001
Swelling at injection site	301 (55.7%)	239 (44.3%)	209 (34.3%)	401 (65.7%)	<0.001
Redness at injection site	91 (16.9%)	449 (83.1%)	80 (13.1%)	530 (86.9%)	0.075
Lymphadenopathy	247 (45.7%)	293 (54.3%)	161 (26.4%)	449 (73.6%)	<0.001
Fever (temperature >37.8 ˚C)	287 (53.1%)	253 (46.9%)	236 (38.7%)	374 (61.3%)	<0.001
Headache	222 (41.1%)	318 (58.9%)	161 (26.4%)	449 (73.6%)	<0.001
Nausea	72 (13.3%)	468 (86.7%)	6 (1.0%)	604 (99.0%)	<0.001
Rashes	356 (65.9%)	184 (34.1%)	187 (30.7%)	423 (69.3%)	<0.001
Burning at injection site	388 (71.9%)	152 (28.1%)	192 (31.5%)	418 (68.5%)	<0.001
Flu	165 (30.6%)	375 (69.4%)	90 (14.8%)	520 (85.2%)	<0.001
Anxiety	208 (38.5%)	332 (61.5%)	146 (23.9%)	464 (76.1%)	<0.001
Muscle pain (myalgia)	361 (66.9%)	179 (33.1%)	178 (29.2%)	432 (70.8%)	<0.001
Fatigue	276 (51.1%)	264 (48.9%)	122 (20.0%)	488 (80.0%)	<0.001
Joint pain	312 (57.8%)	228 (42.2%)	148 (24.3%)	462 (75.7%)	<0.001
Chills	280 (51.9%)	260 (48.1%)	144 (23.6%)	466 (76.4%)	<0.001
Cough	86 (15.9%)	454 (84.1%)	64(1 0.5%)	546 (89.5%)	0.006
Swelling of glands	358 (66.3%)	182 (33.7%)	176 (28.9%)	434 (71.1%)	<0.001
Sore throat	116 (21.5%)	424 (78.5%)	80( 13.1%)	530 (86.9%)	<0.001
Shortness of breath	252 (46.7%)	288 (53.3%)	195 (32.0%)	415 (68.0%)	<0.001
Diarrhea	198 (36.7%)	342 (63.3%)	87 (14.3%)	523 (85.7%)	<0.001
Chest pain	289 (53.5%)	251 (46.5%)	129 (21.1%)	481 (78.9%)	<0.001

The majority of the patients with DM (n=345, 63.9%) and non-DM participants (n=312, 51.1%) were pleased with their vaccination. While only 12 (2.2%) DM patients and 14 (2.3%) non-DM participants were dissatisfied, there was a significant association observed between them (p<0.001), as shown in Table 4.

**Table 4:** Association of level of satisfaction in the DM and non-DM groups DM: diabetes mellitus; COVID-19: coronavirus disease 2019

Variables		DM Group, n (%)	Non-DM Group, n (%)	p-value
Overall subject level of satisfaction with the COVID-19 vaccine	Very satisfied	67 (12.4%)	126 (20.7%)	<0.001
	Satisfied	345 (63.9%)	312 (51.1%)	
	Don’t know	116 (21.5%)	158 (25.9%)	
	Dissatisfied	12 (2.2%)	14 (2.3%)	

## Discussion

Patients with chronic illnesses, such as diabetes, can dramatically lower their risk of COVID-19 by receiving the SARS-CoV-2 vaccine. After receiving the SARS-CoV-2 vaccine, DM patients were not reported to experience any severe side effects in the published studies [18]. This study demonstrated that both diabetes and non-diabetic groups experienced the side effects following COVID-19 vaccination.

One cross-sectional analytical study was conducted in Islamabad on 205 people, in which the participants received 0.5 ml of the COVID-19 vaccination per dosage. Fever was one of the post-vaccination side effects reported by 69 individuals, and 56 had pain, redness, and swelling at the injection site. Chills and stiffness were observed by 42 participants, whereas abdominal distress and flu-like symptoms were recorded by 55 and 28 people, respectively. In comparison to older participants, younger people were more likely to experience abdominal distress and flu-like complaints after vaccination. The emergence of post-vaccination side effects was also strongly related to the clinical history of comorbidities. For instance, diabetes subjects had a higher likelihood of post-vaccination tiredness/malaise (p=0.049) and flu-like symptoms (p=0.013) than non-DM participants [19]. Our study was not in accordance with the above findings and revealed that burning at the injection site followed by fever were the most prevalent side effects after the first dose reported by 455 (84.3%) and 385 (71.3%) patients with DM, while these symptoms were observed in 252 (41.3%) in 359 (58.9%) in the non-DM group, with a significant difference among them (p<0.001).

Similarly, Abbas et al.'s study from Islamabad reported that the Sinopharm vaccination was well received across all age categories (23-55 years) and had no serious side effects. Around 45.4% (93/205) of the patients reported tiredness or malaise as the most frequent side effect following immunization, followed by 39.5%(81/205) headache or migraine [19]. Sinopharm, a comparable vaccination, was evaluated in China; it did not report any tiredness, headache, fever, or muscle pain, but it did report 14.3% regional pain and 2.4% fever [20]. Our study was inconsistent with the above-mentioned research and showed that the most commonly administered vaccine was Pfizer in 187 (34.6%) DM patients and Sinovac in 234 (38.4%) non-DM participants. In terms of side effects, the most commonly observed side effects in patients with DM had burning at the injection site (n=455, 84.3%), fever (n=385, 71.3%), swelling (n=363, 67.2%), and pain (n=349, 64.6%), whereas, in the non-DM group, fever (n=359, 58.9%) was followed by pain at the injection site (n=284, 46.6%). 

Likewise, adverse effects were observed in a clinical study with AstraZeneca that was performed in the UK. Of the participants, 70% expressed tiredness, 68% reported headache, 60% reported muscular pain, and 51% reported feeling fever [21]. Another trial using BioNTech-Pfizer was performed in the United States. Of the subjects, 83.3% felt post-vaccination tiredness, 100% had headaches and regional pain, 58.3% complained of muscle pain, and 66.7% had a fever [22]. Our study indicated that 100 (18.6%) AstraZeneca and 187 (34.6%) Pfizer vaccines were administered in the DM group and 111 (18.2%) and 115 (18.7%) in the non-DM group, respectively. This was in contrast to the above-mentioned studies. Following the first dose, 190 (35.2%) DM patients reported fatigue, 263 (48.7%) headaches, 374 (69.3%) muscular pain, and 385 (71.3%) fever.

Similarly, a different study discovered that 40% of the subjects had at least one side effect following their first COVID-19 vaccine. The Covishield vaccine (AstraZeneca) was administered to more than 91% of the responders, and the most frequent side effects were pain at the injection site, followed by the tenderness of the administered arm (78.9%), fatigue (71.1%), fever (54.9%), and headaches (49.8%). The majority of the early immunization-related symptoms were minor and were gone within three days [23]. Similar to this, studies conducted in India found a prevalence of adverse effects among healthcare workers ranging from 40-70% [24-26]. Additional research in India and other Asian nations has shown that the COVID-19 vaccine frequently causes fever, weariness, muscular discomfort, joint pain, and headaches [27-29]. The current study was not in accordance with the above-reported research and found that AstraZeneca was the least administered vaccine, given to 100 (18.6%) DM and 111 (18.2%) non-DM participants. Burning at the injection site was the most frequent side effect after receiving the first dose, whereas the group with diabetes also experienced fever, muscular pain, and swelling at the injection site.

Interestingly, one cross-sectional survey conducted in China explored the occurrence of SARS-CoV-2 vaccine reluctance amongst diabetes patients. total of 56.4% (273/483) of the 483 participants lacked vaccine confidence, including 41.8% (114/273) who were hesitant and 158.2% (159/273) who were confused. According to the survey of those who were hesitant to receive the SARS-CoV-2 vaccine, their greatest concern about the vaccine's safety and potential side effects was identified [30]. These findings were quite similar to the current study and showed that approximately 345 (63.9%) DM patients and 312 (51.1%) of non-DM participants were pleased with their immunizations and indicated no reluctance to vaccinate themselves. On the other hand, the presence of adverse effects led to dissatisfaction in 12 (2.2%) DM and 14 (2.3%) non-DM participants.

This study had a few limitations. Firstly, the study didn’t compare the efficacy of the COVID-19 vaccine with the natural immunity that is acquired after a COVID-19 infection, as it has been seen in many studies that natural immunity given by COVID-19 was more protective and lasts for a longer duration and without side effects of clotting [31-33]. Conversely, COVID-19 infection can lead to more serious complications like clotting, myocarditis, myocardial infarction, stroke, etc, and on the other hand, adverse side effects (like clotting and stroke) of COVID-19 vaccination remains extremely rare, signifying the protective role of COVID vaccination. Secondly, the participants' motivation or capacity to reply to the survey may have been impacted by the occurrence of adverse effects. Finally, we suggest that the absence of serious adverse effects right after immunization does not necessarily indicate long-term vaccine safety. The validity and dependability of qualified medical experts' assessments of self-reported side effects is the study strength. To improve the pertinent knowledge of patients with diabetes and make more informed decisions about vaccination behavior, the health sector must continue to look into the long-term immunological response of DM patients to the COVID-19 vaccination in the future.

## Conclusions

This study concluded that DM is a prevalent comorbidity in COVID-19-infected individuals, and patients with DM report more side effects from immunization than non-DM participants. The most commonly reported side effects of the vaccine in DM patients were observed to be burning at the injection site, fever, muscle and joint pain, and swelling at the injection site. Additionally, participants with and without DM reported feeling satisfied with their vaccines. Consequently, to improve the pertinent knowledge of patients with DM and make more informed decisions about vaccination behavior, the health sector must continue to look into the long-term immunological response of patients with DM to COVID-19 vaccination in the future.
